# Microalgae Biotechnology: Methods and Applications

**DOI:** 10.3390/bioengineering11100965

**Published:** 2024-09-26

**Authors:** Xianmin Wang, Songlin Ma, Fantao Kong

**Affiliations:** MOE Key Laboratory of Bio-Intelligent Manufacturing, School of Bioengineering, Dalian University of Technology, Dalian 116024, China; xmwang@mail.dlut.edu.cn (X.W.); msonglin@mail.dlut.edu.cn (S.M.)

## 1. Introduction

Microalgae are regarded as sustainable and promising chassis for biotechnology due to their efficient photosynthesis and ability to convert CO_2_ into valuable products [1,2]. In addition, microalgae can also be used to remediate waste resources and pollutants, making them useful for environmental remediation while producing valuable compounds [3]. However, the low product titers from microalgae and high cost of microalgal cultivation limit the economic feasibility of industrial production and pollutant remediation using microalgae [1,4]. Therefore, advancements in microalgal biotechnology are essential to overcome current limitations and meet the increasing demand for commercial applications.

In the past decade, research in microalgal biotechnology has experienced a rapid resurgence, particularly with the advent of disruptive technologies that hold promise for enabling the commercial production of bioproducts [1,4,5,6]. The development of genetic tools and techniques, such as novel transformation methods, vector toolkits, genome editing, and high-throughput screening, is fundamental to advancing microalgal biotechnology [5,7,8]. Furthermore, efficient and innovative bioengineering and biotechnology strategies are essential to reduce production costs in large-scale microalgae cultivations [9,10]. Overall, microalgal biotechnology holds immense potential to enhance microalgae as cell factories for the simultaneous overproduction of valuable biomolecules, remediation of environmental pollutants, and achievement of carbon neutralization. The papers published in this Special Issue reported representatives of recent advancements in genetic engineering tools and techniques applicable to various microalgal species. In addition, the innovative strategies for achieving efficient and cost-effective microalgae cultivation and production were also highlighted.

## 2. Synthetic Biology Facilitates Antimicrobial Peptide Production in Microalgae

Synthetic biology provides a powerful toolkit to enhance natural antimicrobial peptide (AMP) production in microalgae as well as engineer novel AMPs with improved properties [11]. This can also help address the growing need for new antimicrobials to combat antibiotic resistance. It was reported that the green microalga *C. reinhardtii* can be utilized to produce antimicrobial peptides such as taraxacum officinale antimicrobial peptide 4, Bacteriocin LS2, and Mytichitin-CB, which can effectively inhibit the growth of both Gram-negative and Gram-positive bacteria [12,13,14]. These findings indicate that microalgae can serve as a viable platform for the production of active antimicrobial peptides. 

Anti-lipopolysaccharide factors (ALFs) are AMPs with lipopolysaccharide-binding domains that exhibit broad-spectrum antimicrobial activity and hold significant potential for the aquaculture industry [15,16]. Recently, Ou et al. reported that ALFs can be produced and secreted into the extracellular compartment in microalgae through expression of anti-lipopolysaccharide factor 3 (*ALFPm3*) from *P. monodon* that fused to the signal peptide Ice-binding protein 1 (IBP1) derived from a psychrophilic Antarctic alga [17]. Furthermore, they found that transgenic algae successfully synthesized ALFPm3 and secreted it into the culture media, effectively inhibiting the growth of common aquaculture pathogenic bacteria, such as *Vibrio harveyi*, *Vibrio anguillarum*, *Vibrio alginolyticus*, and *Vibrio parahaemolyticus* [17]. Compared to the traditional intracellular expression of AMPs, which requires protein purification through ultrasonic lysis and interrupts continuous production, extracellular expression streamlines the isolation of exogenous gene products in this study. Noticeably, the protein extracts from the culture media of transgenic algal cells showed a higher inhibition rate compared to ampicillin [17]. This superior bacterial inhibition efficacy highlights the exceptional performance of the ALFPm3 produced in microalgae. 

Microalgae engineered to produce AMPs can be used to develop new antimicrobial therapies, providing one solution for the growing issue of antimicrobial resistance (AMR). These peptides can target drug-resistant pathogens, offering a novel approach to combat infections [18]. Moreover, microalgae-based systems for AMP production are environmentally friendly, utilizing light and CO_2_ as primary inputs. This sustainable production method reduces reliance on traditional agricultural resources and minimizes environmental impact [4]. In addition, the application of microalgae as production platforms for AMPs can lower production costs compared to conventional methods. The scalability of microalgal cultivation and the potential for high-yield production make this approach economically viable [4,11]. Taken together, synthetic biology is revolutionizing the production of AMPs in microalgae, employing advanced genetic tools, and leveraging the extracellular expression system. These advancements hold significant potential for therapeutic applications and sustainable production methods.

## 3. Advancements in Microalgae as Novel Chassis for Biotechnology

Microalgae have increasingly been recognized as promising novel chassis for biotechnological applications due to their unique attributes and potential benefits [19,20]. Recent advancements in synthetic biology and genetic engineering have significantly enhanced our ability to manipulate microalgal genomes, enabling their use as efficient biofactories [21]. The state-of-the-art technologies such as CRISPR/Cas9 systems, improved transformation methods, and gene stacking techniques have expanded the genetic toolbox available for microalgae [5,7,21], facilitating the optimization of photosynthetic efficiency, carbon fixation, and metabolic pathways. These advancements have led to improved growth rates and productivity, making microalgae attractive candidates for sustainable production of biofuels, high-value compounds, and recombinant proteins [22]. Additionally, the fact that microalgae possess the ability to utilize atmospheric CO_2_ and grow in non-arable lands underscores their potential for environmental sustainability.

Among microalgae, the model eukaryotic microalga *Chlamydomonas reinhardtii* is a genus of unicellular microalgae that is widely studied in the fields of cell biology, genetics, and biotechnology [23,24,25]. The genome of *Chlamydomonas reinhardtii* has been fully sequenced, which has facilitated precise genetic modifications and functional genomics studies [24]. Tools such as CRISPR/Cas9 and RNA interference are routinely used to study gene function in this alga [5,19]. *Chlorella* sp. and *Spirulina* sp. dominate the global microalgal biomass production, constituting over 90% of the total output [20]. These species are regarded as promising aquaculture-based bioeconomy systems due to their nutritional and therapeutic properties. These microorganisms are poised for significant market expansion, particularly in the nutraceutical, food, and beverage industries [26,27]. Recently, Abreu et al. summarized a comprehensive review of the current and potential applications of *Chlorella* sp. and *Spirulina* sp. [20]. Current commercial applications of *Chlorella* sp. and *Spirulina* sp. mainly include human food and nutrition supplements, aquaculture and agriculture, production of pharmaceutical drugs, cosmetics, and skin care [20,28]. Emerging and innovative applications of *Chlorella* sp. and *Spirulina* sp. mainly include wound-healing dressings, tissue engineering and cancer therapies, microrobots for drug delivery, degradable biopolymer production, and bioremediation [20]. However, to realize the full potential of these microalgae requires addressing significant economic and technological hurdles, as well as specific market concerns. A primary challenge lies in the high costs associated with large-scale cultivation, necessitating a focus on improving cost-effectiveness to achieve wider market adoption. 

Extremophile microalgae, which thrive in extreme environmental conditions, have garnered significant interest due to their unique adaptations and potential biotechnological applications [29]. The snow alga *Sanguina nivaloides* (*S. nivaloides*), mainly distributed in high mountains and polar regions, has evolved to survive and thrive in freezing temperatures. It possesses specialized enzymes and metabolic pathways that remain active at low temperatures, allowing it to photosynthesize and grow in harsh environments [30]. The high levels of astaxanthin produced by *S. nivaloides* have attracted interest for commercial exploitation. Advanced imaging techniques, including X-ray tomography and focused-ion-beam scanning electron microscopy, combined with physicochemical and physiological analyses, have unveiled adaptive features that explain the possible reasons for *S. nivaloides* to thrive in snow environments [30]. Noticeably, the formation of cytosolic droplets in *S. nivaloides* might act as carbon reservoirs (triacylglycerol and carotenoids) and defense against oxidative stress under harsh conditions [30]. Recently, a novel extremophile microalga *Chlamydomonas pacifica* has been explored for their potential in sustainable biofuel production due to their ability to accumulate high levels of lipids and starch without compromising growth [31]. Genetic engineering efforts have been made to further enhance lipid and starch yield through the expression of the *Dof* transcription factor and phosphoglucomutase 1, respectively. The scalability of these engineered strains of *Chlamydomonas pacifica* was also demonstrated by cultivating them in pilot-scale raceway ponds [31]. However, research into the genetics and metabolism mechanisms of these extremophile microalgae is still unclear. Therefore, studies that focus on understanding the genetic/metabolic mechanisms that enable these microalgae to survive and function in harsh environments, such as high salinity, extreme temperatures, and acidic or alkaline conditions, are needed. 

## 4. Innovations in Cost-Effective Microalgae Cultivation Techniques and Applications

Innovations in cost-effective microalgae cultivation techniques are crucial for making microalgal products economically viable and competitive in various markets [32]. These innovations focus on optimizing culture medium, manipulating the illumination time, developing advanced photobioreactors, and improving harvesting techniques [33]. 

Recently, Kuo et al. found that when *Chlorella* sp. was cultivated using 20 g/L fertilizer medium for 6 days, the biomass productivity and lutein content were 1.04 g/L/d and 4.41 mg/g, which were higher (>1.3-fold) than when cultivated in modified f/2 medium [34]. In addition, a 97% decrease in medium cost per gram of microalgal biomass was found using this strategy. Furthermore, the optimized 20 g/L fertilizer medium by adding 20 mM urea increased lutein content to 6.03 mg/g, and a 96% reduction in medium cost per gram of lutein was achieved [34]. Noticeably, microalgal lutein produced under these organic fertilizers with urea supplements significantly reduced reactive oxygen species levels in mammal NIH/3T3 cells exposed to blue-light irradiation [34]. This study demonstrates the effectiveness of optimized culture medium in enhancing *Chlorella* sp. cultivation and lutein production while significantly reducing costs. The produced lutein shows promise in protecting against blue-light-induced oxidative stress, suggesting potential applications in various industries.

Photobioreactors (PBRs) are essential for the efficient cultivation of microalgae, offering various designs to optimize growth conditions and productivity [35]. The most commonly used photobioreactors include raceway ponds, tubular photobioreactors, flat plate photobioreactors, and bubble column photobioreactors [10]. Light is also a critical factor that significantly influences microalgal growth and productivity. Microalgae, being photosynthetic organisms, rely on light as their primary energy source for carbon fixation and biomass production [36,37]. The effects of light on microalgal growth are multifaceted. Light-emitting diodes (LEDs) are used as artificial light sources in *Haematococcus pluvialis* (*H. pluvialis*) cultivation due to their energy efficiency [38]. Immobilized cultivation of *H. pluvialis* was performed in pilot-scale angled twin-layer porous substrate photobioreactors (TL-PSBRs). Increasing illumination time to a 22/2 h light/dark cycle with red and blue LEDs at 120 µmol photons m^−2^ s^−1^ resulted in 7.5 g m^−2^ day^−1^ biomass productivity and 2% astaxanthin in dry biomass [38]. Furthermore, 10–40 mM NaHCO_3_ addition caused a high percentage of astaxanthin accumulation in dry weight during the first four days in TL-PSBRs, while 30–80 mM NaHCO_3_ addition inhibited algal growth and astaxanthin accumulation [38]. These studies demonstrated the importance of optimizing light cycles and precisely managing inorganic carbon concentrations to enhance biomass productivity and astaxanthin accumulation in *H. pluvialis* cultivation using TL-PSBRs. Increasing the illumination time with combined red and blue LEDs can increase the biomass and stimulate the astaxanthin accumulation of *H. pluvialis* in pilot-scale angled TL-PSBRs. 

The combined use of industrial wastewater for microalgae cultivation in photobioreactors presents a sustainable and cost-effective approach for circular bioeconomy [39]. This method leverages the nutrient-rich nature of wastewater to support the growth of microalgae, which can then be harvested for biofuels and other valuable bioproducts [40,41]. Recently, Abraham et al. investigated the feasibility of algal growth in the target wastewater on a larger outdoor scale [42]. Two 1000 L open raceway ponds were used in a greenhouse setting to test algal growth in industrial wastewater, and an online system was used to track operational parameters, such as temperature, pH, light intensity, and dissolved oxygen [42]. In this culture system, the highest average areal biomass productivity was 23.9 g/m^2^d during summer, with a biochemical methane potential (BMP) of 350 scc/gVS [42]. Under low nitrogen conditions, oil content of 22 wt.% was observed. Economic and environmental analysis showed the viability of the wastewater valorization approach and helped optimize process performance for future scale-up [42]. Therefore, cultivating microalgae using industrial wastewater offers a promising pathway for sustainable bioeconomy. It combines wastewater treatment with biomass generation, providing economic and environmental benefits. However, further research and technological advancements are essential to overcome existing challenges and fully realize the potential of this innovative approach.

## 5. Conclusions

Microalgae are noted for their versatility in producing bioactive molecules, biochemicals, and biofuels. Their ability to utilize atmospheric CO_2_ makes them a sustainable feedstock for these valuable products. The potential of microalgae in pollutant removal and waste resource remediation offers a dual benefit of environmental remediation and the production of value-added products. Despite their potential, the industrial production of microalgal products faces significant economic and biotechnological constraints. High cultivation costs and low product yields are major hurdles that need to be addressed. Advancing microalgal biotechnology to overcome these challenges is still urgently needed. Recent developments in genetic engineering tools, such as CRISPR/Cas9, novel transformation methods, and high-throughput screening techniques, are fundamental to this progress. Efficient bioengineering and innovative cultivation strategies are essential to reduce production costs and make large-scale microalgae cultivation economically viable.
